# A Bioprinted Heart-on-a-Chip with Human Pluripotent Stem Cell-Derived Cardiomyocytes for Drug Evaluation

**DOI:** 10.3390/bioengineering9010032

**Published:** 2022-01-13

**Authors:** Alan Faulkner-Jones, Victor Zamora, Maria P. Hortigon-Vinagre, Wenxing Wang, Marcus Ardron, Godfrey L. Smith, Wenmiao Shu

**Affiliations:** 1 Institute of Mechanical, Process and Energy Engineering, School of Engineering and Physical Sciences, Heriot-Watt University, Edinburgh EH14 4AS, UK; Alan.Faulkner-Jones@hw.ac.uk (A.F.-J.); isaacwenxing@gmail.com (W.W.); 2 Departamento de Ingeniería Mecánica, Energética y de los Materiales, Universidad de Extremadura, 06006 Badajoz, Spain; victor@unex.es; 3 Departamento de Bioquímica y Biología Molecular y Genética, Universidad de Extremadura, Facultad de Ciencias, 06006 Badajoz, Spain; 4 Renishaw PLC, Research Avenue North, Edinburgh EH14 4AP, UK; Marcus.Ardron@Renishaw.com; 5 Institute of Cardiovascular and Medical Sciences, University of Glasgow, Glasgow G12 8QQ, UK; Godfrey.Smith@glasgow.ac.uk; 6 Clyde Biosciences, Glasgow G12 8QQ, UK; 7 Department of Biomedical Engineering, Faculty of Engineering, University of Strathclyde, Glasgow G4 0NW, UK; will.shu@strath.ac.uk

**Keywords:** cell-printing, organs-on-a-chips, bio-ink, hydrogels, hiPSC-CMs, in vitro testing, drug screening

## Abstract

In this work, we show that valve-based bioprinting induces no measurable detrimental effects on human-induced pluripotent stem cell-derived cardiomyocytes (hiPSC-CMs). The aim of the current study was three-fold: first, to assess the response of hiPSC-CMs to several hydrogel formulations by measuring electrophysiological function; second, to customise a new microvalve-based cell printing mechanism in order to deliver hiPSC-CMs suspensions, and third, to compare the traditional manual pipetting cell-culture method and cardiomyocytes dispensed with the bioprinter. To achieve the first and third objectives, iCell^2^ (Cellular Dynamics International) hiPSC-CMs were used. The effects of well-known drugs were tested on iCell^2^ cultured by manual pipetting and bioprinting. Despite the results showing that hydrogels and their cross-linkers significantly reduced the electrophysiological performance of the cells compared with those cultured on fibronectin, the bio-ink droplets containing a liquid suspension of live cardiomyocytes proved to be an alternative to standard manual handling and could reduce the number of cells required for drug testing, with no significant differences in drug-sensitivity between both approaches. These results provide a basis for the development of a novel bioprinter with nanolitre resolution to decrease the required number of cells and to automate the cell plating process.

## 1. Introduction

The pharmaceutical industry faces many challenges in the development of new drugs. It can take decades to successfully develop a novel drug and only a very small number of drug candidates are approved for human use since most drug candidates (90%) fail in the last stage (clinical trials), mainly due to safety issues [1,2,3,4]. The most common causes of drug candidates’ failure and drug attrition are unexpected toxicity issues, specifically hepatotoxicity and cardiotoxicity: 45% of the total post-approval drug withdrawals are due to cardiovascular liabilities, and 32% of the failures are due to hepatotoxicity [5]. Therefore, the use of human biological models during preclinical assays is key to fill the gap found when classical animal models are employed.

The majority of current research involving the evaluation of cardiovascular physiology involves studies using traditional animal models [6]. It is well-known that there are metabolic, anatomic and cellular differences between humans and these animals; for example, the average resting heart rate of humans is 60–100 bpm while this can be very different in animal models, such as murine (500–700 bpm), canine (80–120 bpm), porcine (70–120 bpm) and primate (160–330 bpm) models [6,7,8,9,10]. Despite this, non-human animals are the most common models used for drug testing due to the difficulties of accessing human sample tissues. In this sense, human-induced pluripotent stem cell-derived cardiomyocytes (hiPSC-CMs) are an excellent alternative to animal models because they express human cardiac ion channels in a native environment and have proved to be a good alternative to the classical animal models for drug screening, thus avoiding interspecies variabilities [11,12,13]. The main issues when using hiPSC-CMs lie in the high technological complexity of generating the cells and the high cost of commercial lines. Therefore, approaches aimed at reducing the number of cells needed for drug-testing is key to reduce the cost of the assay and allow the widespread adoption of this cell model across the pharmaceutical industry.

Traditional two-dimensional cell-culture methods have limited similarity to in vivo tissues [14,15,16,17] and spheroid models have practical problems [18,19,20] as well as geometric constraints. Several technologies are being developed to better represent in vivo tissues including organs-on-chips [14,21,22,23,24] and bioprinted tissues [25,26,27,28]. This latter type of cell deposition technique enables a reduction in the number of cells used, since we can demarcate a minimum area of bioprinted cells, exactly where they are needed, which results in less cells required per assay. This reduction in the number of cells is especially important with hiPSC-CMs, a costly model from both a manufacturing and monetary point of view. In the case of high-throughput drug screening techniques, the reduction in the number of cells needed per assay will reduce the cost of this step, which is critical in the preclinical phase of drug discovery [12].

The employment of bioprinting technologies allows deposition of the cells and biomaterials to generate complex tissue-like structures with functional similarity to in vivo tissues. Despite this significant advantage, bioprinting is not a harmless procedure for cells as the setup can expose cells to mechanical and/or thermal stresses, which can affect their physiological performance due to the shear stress [29,30,31].

In the 3D biofabrication process, the two key components are the bioprinter and the bio-ink. The materials used as bio-ink include natural and synthetic polymers [32]. Alginates, which are natural polysaccharides, are one of the most common bio-ink materials employed during 3D bioprinting processes because they are printable, inexpensive, biocompatible, and biodegradable. The last two characteristics are critical for in vivo applications [32,33]. These hydrogels are prepared in water solutions and do not exhibit flow in the steady state, providing physical properties comparable to human tissues. The gelling process of alginates requires the presence of multivalent cations such as Ca^2+^ [34,35]. The viscosity of the hydrogel will depend on the relative proportions of the alginate/crosslinker present; therefore these proportions should be carefully controlled since high viscosity can affect the dispensing process [35]. The main disadvantage of alginates is their low cellular adhesion, which compromise the cell culturing process; this issue can be solved by including adhesion peptides in the specific case of cardiac bioprinting. An additional challenge exists since electrical cell-to-cell coupling is crucial for cellular function; therefore, a bio-ink that is able to maintain the conductivity of the tissue is essential [32].

In this paper, we conducted a detailed investigation to design a user-friendly bioprinter setup that minimally affects the hiPSC-CMs’ physiology. At each stage, the novel process was compared to the traditional approach (manual pipetting) to ensure the reliability of the results. Firstly, the electrophysiological behaviour of hiPSC-CMs plated using bioprinting technique and common hydrogels and their precursor solutions was compared with traditionally manual-plated cells using common matrix (fibronectin). To demonstrate the suitability of the bioprinting technique for drug-testing evaluation, the hiPSC-CMs were plated using the bioprinter or traditional methodology (manual pipetting) and were treated with three previously well-characterised drugs (E4031, flecainide and nifedipine). The electrophysiology of those cells was evaluated using the high-throughput optical platform CellOPTIQ^®^ (Clyde Biosciences Ltd., Newhouse, Glasgow, UK). The response of bioprinted or manual-plated cells to E4031, nifedipine and flecainide was as expected in both cases, providing further evidence of the utility of the CellOPTIQ^®^ setup. This research serves as the basis of a bioprinting-based strategy in which bioprinting and substrate modification methods are employed to replace traditional manual culture processes for high throughput drug evaluation. This strategy has the advantages of speeding up the plating and testing processes and reducing the cost by drastically decreasing the number of required cells.

## 2. Materials and Methods

### 2.1. Cell Culture

Cryopreserved human-induced pluripotent stem cell-derived cardiomyocytes (hiPSC-CMs) (iCell^2^, Cellular Dynamics International, Madison, WI, USA) were plated following the manufacturer’s instructions in 96-well glass bottom plates (P96G-1.5-5-F, MatTek, Bratislava-Ružinov, Slovakia) coated with fibronectin 1:100 at a final density of 156,250 cells/cm^2^. The cells were used from 7–10 days after initial plating; during this time they were kept in Manufacturer’s Maintenance Medium in a laboratory incubator set at 36.0–37.5 °C, 5.0 ± 0.5% CO_2_ [36].

When hydrogels were used, the same 96-well plates were employed, using the hydrogels as coating instead of fibronectin. For the substrate modification experiments, 35 mm glass-bottomed plates (P35G-0.170-14-14C, MatTek, Bratislava-Ružinov, Slovakia) were used in order to restrict the coating and plating within the modified areas.

### 2.2. Hydrogel Materials

Two hydrogel materials and their respective cross-linkers were procured: sodium alginate (A1112, Sigma-Aldrich Co. LLC, St. Louis, MO, USA) and PRONOVA Ultrapure (PRONOVA UP VLVG, Novamatrix, Sandvika, Norway). In both cases, calcium chloride dihydrate (223506, Sigma-Aldrich Co. LLC, St. Louis, MO, USA) was used as the crosslinker to form alginate hydrogels.

### 2.3. Bioprinting Platform

**Figure 1:**
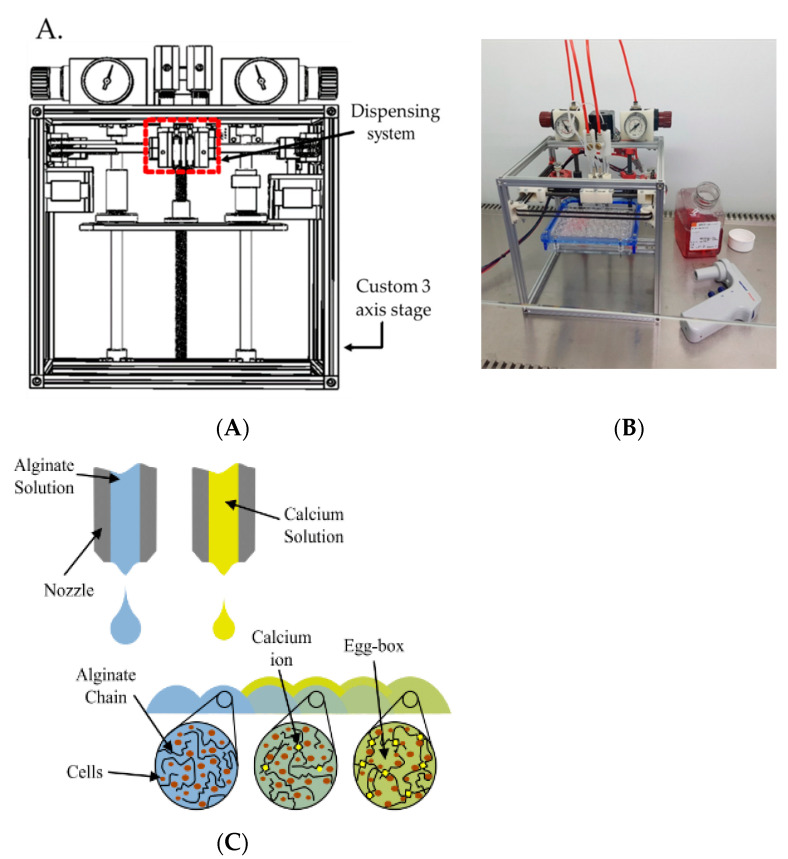
(**A**) Schematic drawing of the cell printer system. (**B**) Bioprinter system within a culture hood, highlighting the small footprint of the machine with a 24-well plate, medium and pipette shown for scale. (**C**) Schematic of the combinatorial printing process for alginate hydrogel creation.

A custom microvalve-based cell printing mechanism has been developed [37,38] that is capable of delivering cell suspensions with nanolitre resolution. This new system has a significantly reduced footprint to suit operation within a biohood (Figure 1). Living cells pass through the delivery system. We previously demonstrated that the delivery process was capable of printing human pluripotent stem cells without affecting their key biological functions, including pluripotency and post-printing differentiation into hepatocyte-like cells with albumin secretion and morphology similar to hepatocytes [38].

Unless otherwise stated, standard printing conditions were used: 2D printing was carried out using a pulse duration of 8 ms with an inlet pressure of 0.6 bar using a nozzle with an internal diameter of 101.6 µm; 3D printing was carried out using a pulse duration of 400 µs with an inlet pressure of 1.0 bar for sodium alginate solution and a pulse duration of 400 µs with an inlet pressure of 0.5 bar for calcium chloride solution, both using nozzles with an internal diameter of 101.6 µm.

### 2.4. Substrate Modification

**Figure 2:**
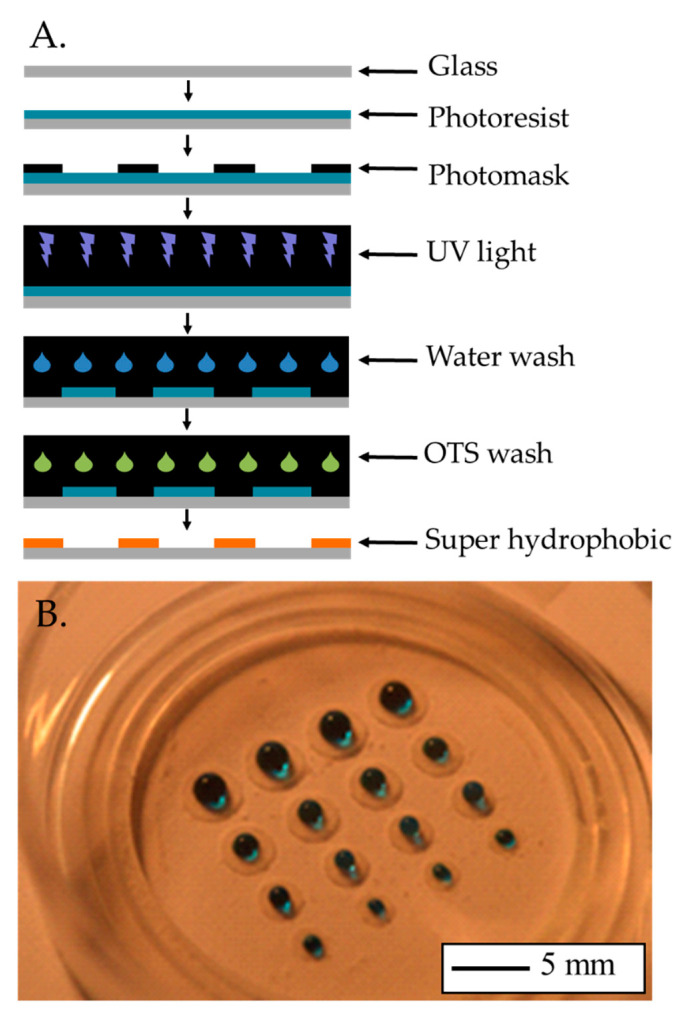
(**A**) Schematic of the substrate modification procedure. (**B**) Photograph of an array of super-hydrophobic rings created with this procedure, water with blue dye shows the hydrophobicity.

To reduce the number of cells required while maintaining cell density, the areas that cells could occupy were reduced by employing traditional microfluidic techniques (Figure 2). Briefly, a layer of Ordyl 940 photo-resistive film (40 μm thick) was applied to a glass-bottomed culture dish (P35G-0.170-14-14C, MatTek, Bratislava-Ružinov, Slovakia). A micro-lithography photo mask defining the required pattern was placed over the top of the film. The 3-layer assembly was exposed to UV light for 2 s and then left to stand for approximately 20 min. The mask was then removed, and the glass culture dish was washed in developer solution, leaving behind the exposed pattern, which was then baked under the UV light for a further 3 min. The glass culture dish was treated with oxygen plasma etching to remove organic contaminants from the surface followed by immediate immersion in deionised (DI) water to form hydroxyl groups. The surface was further treated with 1 mL at 2.5 mM of Octadecyltrichlorosilane (OTS) solution, which was allowed to dry under ambient conditions. The modified surfaces exhibited super-hydrophobic behaviour.

The modified substrate was coated with fibronectin and the hiPSC-CMs were plated following the protocol described in Section 2.1. The cell density was kept to 156,250 cells/cm^2^. The aim of this study was to establish the minimum area able to provide the voltage signal for measuring tissue behaviour.

### 2.5. High Sensitivity Physiological Function Measurement

hiPSC-CMs were plated in glass-bottomed 96-well plates using the bioprinter or a multichannel pipette, method used as a control. The electrophysiology was assessed using the multi-parametric platform CellOPTIQ^®^ (Clyde Biosciences, Ltd., Newhouse, Glasgow, UK) [39,40,41,42] The spontaneous electrical activity was recorded in cells previously loaded transiently with the voltage sensitive dye Di-4-ANEPPS (4 µM, 1 min, room temperature). Intracellular calcium was measured in cells transiently loaded with the fluorescent Ca^2+^ sensor Fura 4f-AM (3 µM, 30 min, 37 °C). Both fluorescence measurements were ratiometric and used a sampling rate of 10 kHz and 0.5 kHz, respectively [43]. The contractility data was recorded on the same platform using a digital camera (ORCA-Flash4.0, Hamamatsu Photonics (UK) Ltd., Welwyn Garden City, UK) to record high resolution images at 0.1 kHz. The baseline electrical activity was recorded from independent wells (200 × 200 µm area, with approximately 25–30 cells in the selected area) for 20 s [36]. Data was analysed offline using CellOPTIQ^®^ software. The absolute values were normalised as percentage change of the baseline.

### 2.6. Cardiotoxicity Screening of Hydrogels and Their Cross-Linkers

Cardiomyocytes were cultured for 7–10 days before being transiently loaded with a voltage-sensitive dye (Di-4-ANEPPS) to measure transmembrane action potential. The cells were then exposed for 30 min to the different sodium alginates, each at a range of concentrations (0.05–2%). The electrical activity was measured (as described in the previous section) and the cell morphology was recorded at 5 and 30 min post-exposure.

The response of cardiomyocytes to the cross-linking solution was also investigated in a similar manner using a range of calcium chloride concentration between 0.2–1%.

### 2.7. Electrophysiology Assessment

On the assay day, the cells were loaded with the fluorescent dyes (Di-4-ANEPPS and Fura 4-AM) in serum-free (SF) media two hours before starting the baseline measurements. The experiments were performed in SF medium. Alginate was added by replacing 100%, 66.67% and 33.33% of the well volume by 1.5% alginate (resulting in alginate concentrations of 1.5%, 1% and 0.5%, respectively). A parallel control set was recorded (negative control) without any alginate. Calcium was added over the alginate substrate and cells. Electrical activity, calcium transient and contractility were measured and analysed as described in the previous section.

### 2.8. Bioprinting of Alginate Barriers for Reduced Cellular Numbers

Bio-ink droplets containing a liquid suspension of live cells and sodium alginate chains were deposited by one printing valve followed by a droplet of a calcium solution from a second valve. Sodium alginate chains become cross-linked by the calcium ions and form a 3D hydrogel network, which encloses the cells and forms a self-supporting structure.

Refinement of this process has led to self-supporting structures up to centimetre scale while utilising the properties of biocompatibility, permeability and biodegradability [19] Sufficient resolution of fluid delivery allows rapid construction on single millimetre scale. These constructs enclose cells of sufficient number and density to be statistically useful and for communications to be established, while avoiding the need for excessive use of costly stem cells, associated culture media, and reagents.

### 2.9. Drug Testing Process Flow Integration and Validation

The drug assay was performed 7–10 days post-thawing in cells plated with traditional manual pipetting or bioprinting. The cells were loaded with the fluorescent dyes (Di-4-ANEPPS and Fura4F-AM, for voltage and intracellular calcium measurements respectively) in SF media. After recording the baseline behaviour for voltage, calcium and contraction, the drugs were added and maintained for 30 min, and the electrophysiological parameters were recorded again. Three well-known drugs were tested: IKr blocker, E4031; L-type Ca^2+^ channel (LTCC) blocker, nifedipine; and Na^+^ channel blocker, flecainde. A set of vehicle control was also used (0.1% DMSO). The number of repeats per condition was *n* = 5. The data were normalised as percentage change of baseline, as previously.

### 2.10. Data Analysis and Statistics

Statistical analysis was performed using Dunnett’s test following ANOVA to allow the comparison of a number of treatments with a single control. Statistical significance was designated as * *p* < 0.05; ** *p* < 0.01; *** *p* < 0.001.

## 3. Results and Discussion

**Figure 3:**
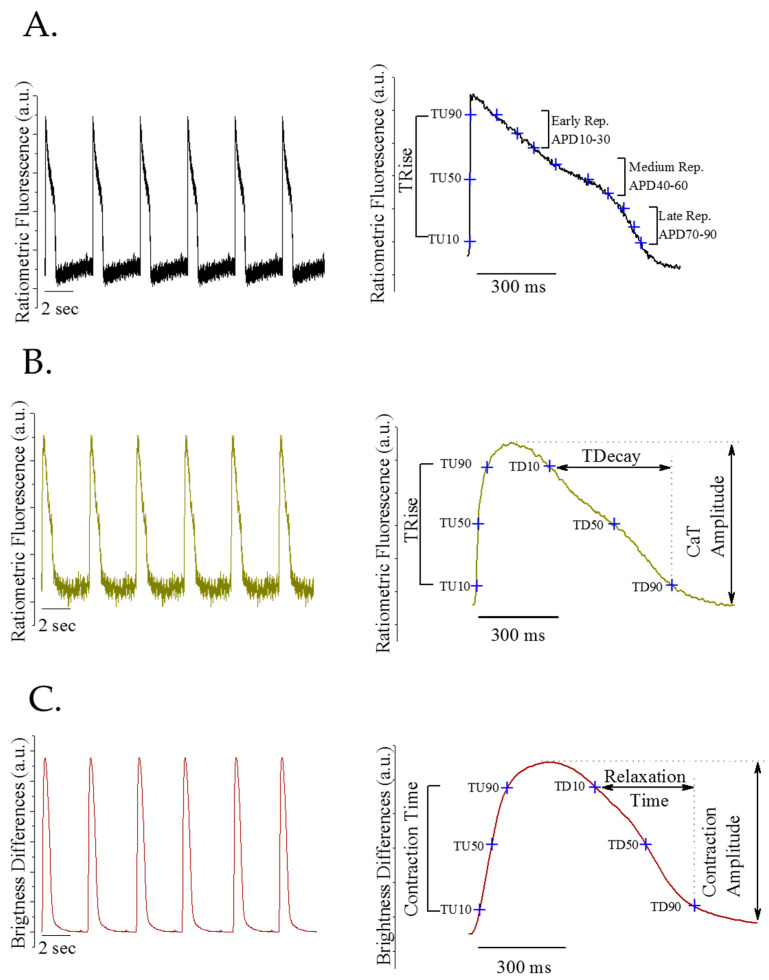
Baseline representative recordings of spontaneous electrophysiological activity iCell^2^ monolayer. (**A**) Action potential traces (left panel), averaged action potential from the recording pointed key parameters (right panel). (**B**) Calcium transient traces (left panel), averaged calcium transient from the recording pointed key parameters (right panel). (**C**) Contraction profile traces (left panel), averaged contraction profile forms the recording pointed key parameters (right panel).

Baseline action potential, calcium transient and the contraction profile from monolayers of spontaneously beating iCell^2^ cardiomyocytes plated by manual pipetting are shown in Figure 3A–C. The panels on the left show a 20 s recording, by way of example. In the panels on the right, the averaged signals from the baseline recordings of the corresponding recorded traces (left panels) are shown and each cardinal cardiac function parameter is indicated.

The voltage measurement provides the depolarisation time (TRise) and the action potential duration (APD) from 10–90% repolarisation. The calcium measurement provides the transient amplitude and the Ca^2+^ release and uptake time (TRise and TDecay, respectively). Finally, the contraction recording shows the contraction amplitude and the contraction and relaxation duration (contraction time and relaxation time, respectively).

**Figure 4:**
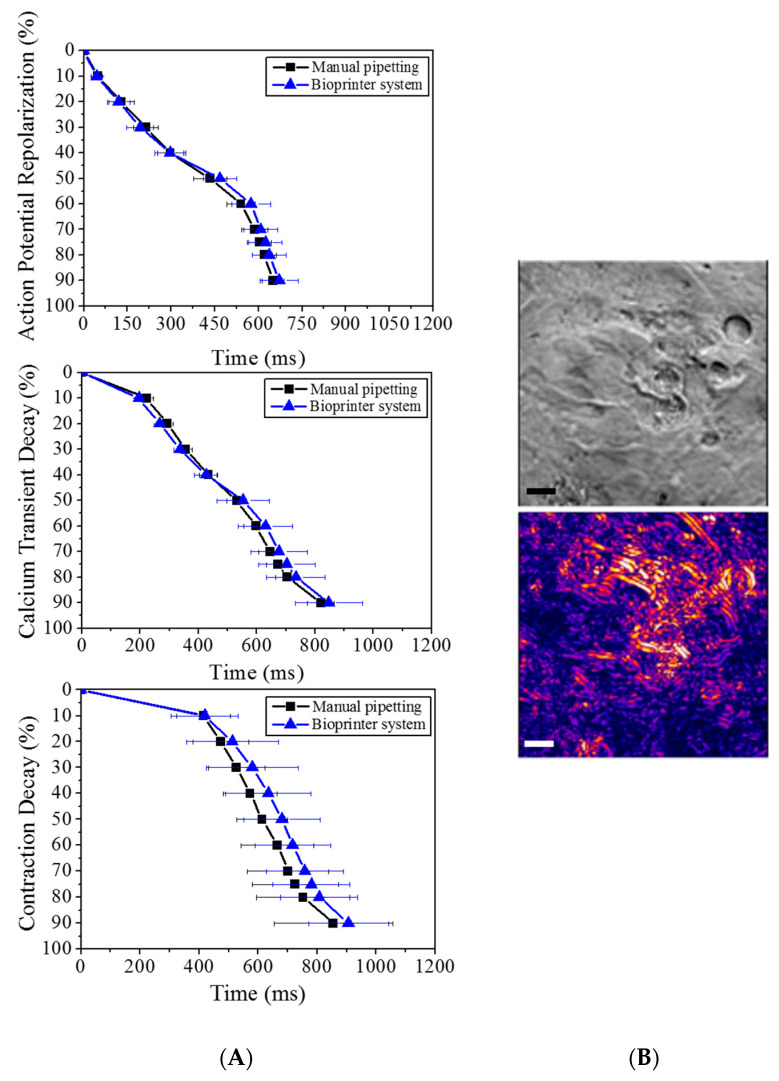

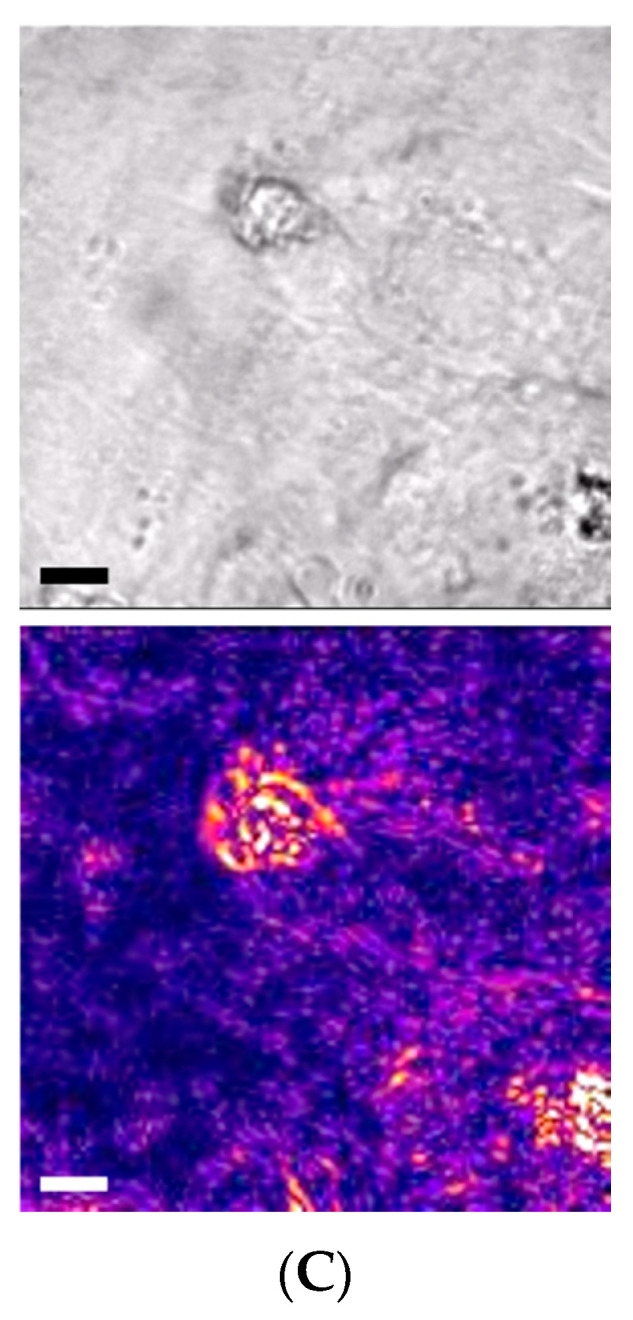
Electrophysiological measurements of 2D tissue constructs delivered by manual pipette (control) or bioprinter valve (**A**). The three parameters assessed are the electrical activity on the cells (action potential), intracellular Ca^2+^ (calcium transient) and mechanical activity (contractile kinetic decay). Contractility images of printed cardiomyocytes, bright-field and computed contractility expressed as brightness: control cells deposited using pipette (**B**) and bioprinted cells (**C**), the scale bar represents 10 microns.

To demonstrate that the printing process causes no measurable detrimental effects to the cells, we compared the electrophysiological behaviour of printed cells with that of the control (manual pipetting). Our findings confirmed that there was no significant statistical difference in any of the three parameters assessed (Figure 4). In addition to showing no significant changes in the baseline electrophysiological behaviour (voltage, intracellular calcium and contraction) of cardiomyocytes plated using the traditional manual pipetting and the bioprinter, both populations were exposed to well-known drugs to dismiss any effect of the printing process on the cardiomyocytes’ sensitivity to cardiotoxic compounds.

**Figure 5:**
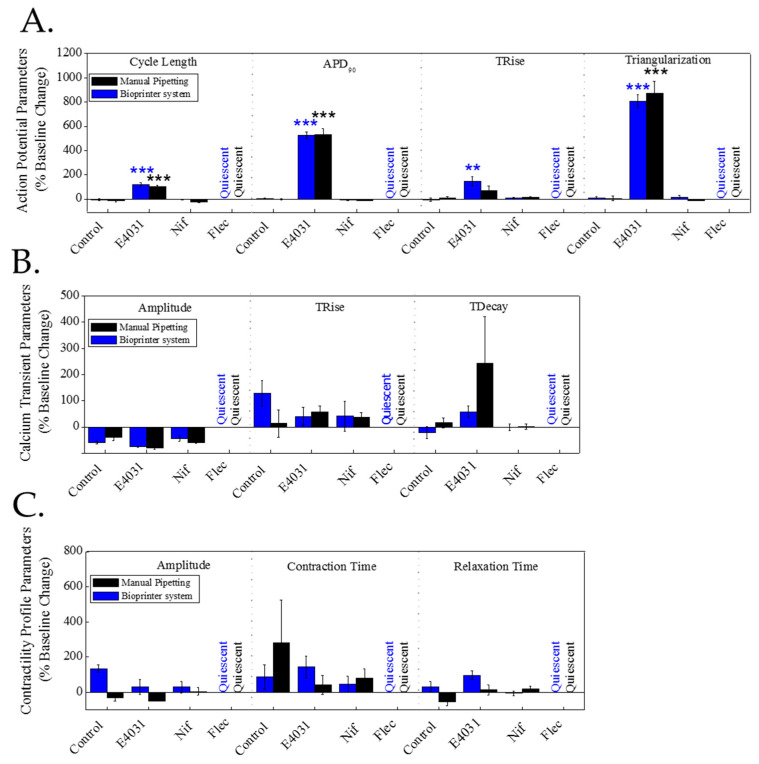
Effect of three well-known cardiotoxic drugs (E4031, nifedipine and flecainide) on hiPSC-CMs electrophysiology cultured using traditional pipetting vs. bioprinter: iCell^2^ hiPSC-CMs cultured in glass-bottomed plates using traditional pipetting (black) or bioprinter (blue) were acutely treated (30 min) with well-known cardiotoxic drugs, the IKR blocker, E4031 (30 nM), the LTCC blocker, nifedipine (10 nM) and the Na^+^ channel blocker, flecainide (1 µM). Electrical activity (**A**), intracellular calcium (**B**) and contraction (**C**) were measured before (baseline) and after 30 min in presence of drugs or vehicle control (0.1% DMSO). The bar graphs show the % change of baseline. Data presented as mean ± SEM (*n* = 5). The statistical analysis was done using the post-hoc Dunnet test ** *p* < 0.01; *** *p* < 0.001.

Figure 5 shows the results as the percentage change of baseline for vehicle control (DMSO 0.1%) and the three compounds tested (E4031 30 nM, nifedipine 10 nM and flecainide 1 µM) of mean ± SEM. No significant differences were observed in drug sensitivity between cells printed or plated with manual pipetting. In the case of the IKr blocker, E4031, the AP was prolonged due to the blockage of K^+^ channels responsible for the repolarisation phase. The LTCC blocker, nifedipine caused the shortening of the AP duration due to the blockage of Ca^2+^ channels, preventing the increase in cytosolic calcium levels. Finally, the Na^+^ channel blocker, flecainide made the cells electrically and mechanically quiescent at the concentration tested due to the blockage of the Na^+^ channels, which are responsible for triggering the AP and therefore the cell contraction. The effects of those three well-known drugs were as expected in both cases, demonstrating that bioprinting of hiPSC-CM does not affect the sensitivity of the cells to the drugs. Parallel effects were reported on key electrophysiological parameters for both of the methods of culturing hiPSC-CM used here.

**Figure 6:**
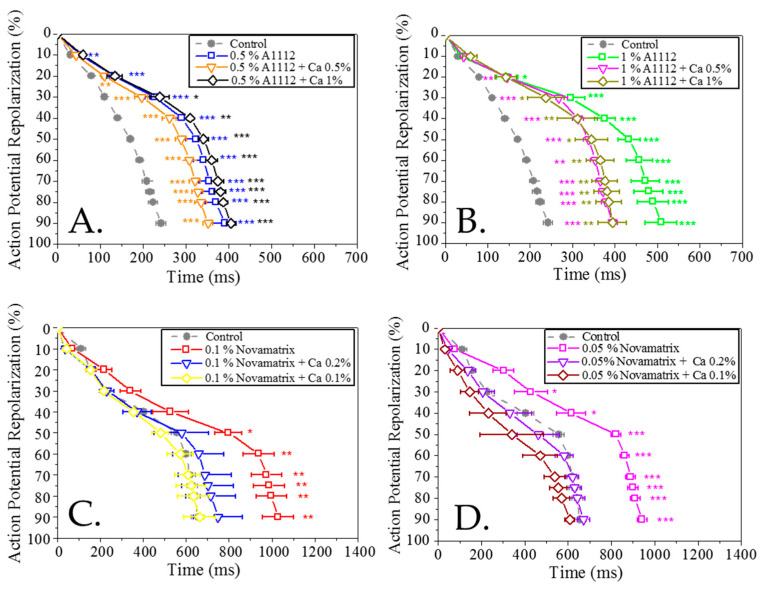
Analysis of the repolarisation profile of cardiomyocytes exposed to varying concentrations of two different alginates (A1112 and Novamatrix) with and without calcium chloride compared to a control: (**A**) 0.5% A1112 and calcium chloride (0%, 0.5%, 1%); (**B**) 1% A1112 and calcium chloride (0%, 0.5%, 1%); (**C**) 0.1% Novamatrix and calcium chloride (0%, 0.1%, 0.2%); (**D**) 0.05% Novamatrix and calcium chloride (0%, 0.1%, 0.2%). Data presented as mean ± SEM (*n* = 5). The statistical analysis was done the post-hoc Dunnet test * *p* < 0.05; ** *p* < 0.01; *** *p* < 0.001.

If alginate hydrogels are to be used with these cells, an in-depth investigation needed to be undertaken to check that their functionality was unaffected by the presence of the alginates and their cross-linkers. At alginate (A1112) concentrations of 0.5% and 1%, an increase in the action potential duration (APD) was observed (Figure 6A,B) and the prolongation showed a dose–dependent effect, being higher at approximately 1%, but in both cases with similar statistical significance. The prolongation of APD started at 20% of the repolarisation phase and continued for the medium and late repolarization phases and there was no significant difference for the other electrophysiological parameters, such as TRise, triangularisation, diastolic interval and cycle length. The addition of calcium to the alginate at two different concentrations (0.5% and 1%) also showed the prolongation of the APD with significant differences for the early, medium and late repolarisation parameters compared to the cells without alginate.

Initial tests with the Novamatrix Ultrapure alginate resulted in a quiescent effect at the same concentrations used to test A1112; therefore, lower concentrations of this material were used. At alginate concentrations of 0.1% and 0.05% an increase in the APD was observed (Figure 6C,D); this is more pronounced for 0.1% but in both cases with significant differences for medium and late repolarisation parameters. The prolongation of APD was clear for the early (20%, 30% repolarisation), medium and late repolarisation parameters. The addition of calcium chloride to the Novamatrix Ultrapure alginate resulted in a shortening of the APD, consistent with the known inverse relationship between extracellular calcium and APD [44]. The CL values were 3.02 ± 0.05 s for the control and alginates and 2.12 ± 0.21 s was the average for the alginate with calcium chloride.

**Figure 7:**
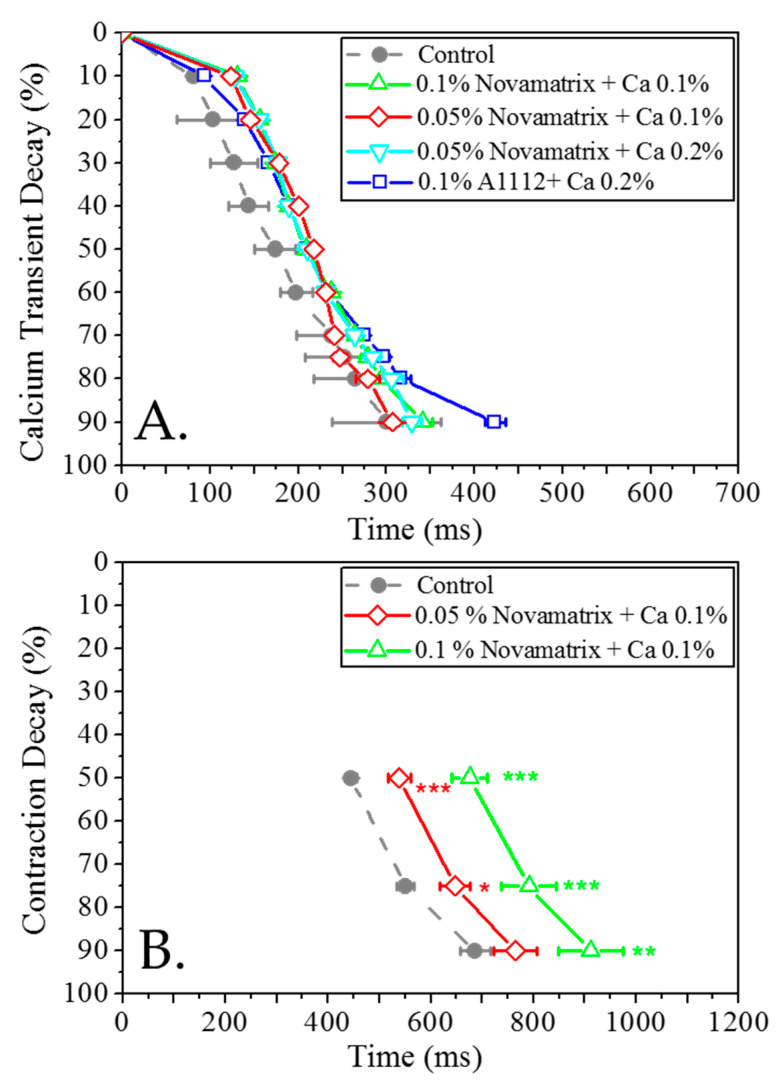
Calcium transient and contractility in cardiomyocytes exposed to alginate and a control: (**A**) calcium transient for various cross-linked Novamatrix alginates; (**B**) contractility for Novamatrix alginate. Data presented as mean ± SEM (*n* = 5). The statistical analysis was done using the post-hoc Dunnet test * *p* < 0.05; ** *p* < 0.01; *** *p* < 0.001.

Analysis of the calcium transient (CaT) revealed no significant differences between both alginate concentrations plus calcium and the control. This is explained by the reduction in the cycle length (CL) due to the increase in the calcium concentration in the cell media (Figure 7A).

Novamatrix alginate concentrations (0.1% and 0.05%) were tested with regard to contractility. A significant increase in the contraction duration (CD) was observed. The prolongation of CD was very clear for medium and late repolarisation parameters (Figure 7B).

The substrate modification experiments, designed to demonstrate the minimum number of cells required to provide voltage signal, showed that the cells plated in the four areas tested (0.1, 0.39, 1.57, 3.53 mm^2^) were able to produce a recordable voltage signal that was clean enough to obtain all the voltage parameters (TRise and APDs) required by electrophysiological studies.

## 4. Conclusions

To the best of our knowledge, this study is the first to demonstrate that the functionality of hiPSC-CMs is adversely affected by the addition of a common hydrogel. Importantly, we also verified that our valve-based printing process has no measurable detrimental effects when compared to conventional manual pipetting and it does not affect the physiological function of hiPSC-CMs. There was no statistically significant difference between cardiomyocytes dispensed with the bioprinter and the standard culture (manual handling) or in their ability to detect cardiotoxicity. A1112 and Novamatrix commercially-available alginates were used. Cardiomyocytes were transiently exposed to alginate and cross-linker precursor solutions in isolation and combination at a range of concentrations and showed detrimental effects when compared to controls. The contractility data show the clear prolongation effect of the repolarisation in the presence of alginate; this effect could be the cause of the blockage of repolarisation K^+^ currents. Interestingly, although the cells were so negatively affected by the addition of calcium alone, experiments to measure intracellular calcium transient did not show a significant difference in function with the addition of calcium in the presence of alginate.

The ability to bioprint hiPSC-CMs while maintaining their physiological function will enable the augmentation of traditional manual processes for novel drug evaluation in order to speed up the testing process, reducing the cost by drastically decreasing the number of cells required, as well as increasing the clinical relevance of these tests by replacing outmoded animal models.

## Data Availability

The data presented in this study are available on request from the corresponding author.
